# Analysis of Cancer Survival Associated With Immune Checkpoint Inhibitors After Statistical Adjustment: A Systematic Review and Meta-analyses

**DOI:** 10.1001/jamanetworkopen.2022.27211

**Published:** 2022-08-17

**Authors:** Emily Pei-Ying Lin, Chih-Yuan Hsu, Lynne Berry, Paul Bunn, Yu Shyr

**Affiliations:** 1 Department of Biostatistics, Vanderbilt University Medical Center, Nashville, Tennessee; 2 Center for Quantitative Sciences, Vanderbilt University Medical Center, Nashville, Tennessee; 3 Division of Pulmonary Medicine, Department of Internal Medicine, School of Medicine, College of Medicine, Taipei Medical University, Taipei, Taiwan; 4 Division of Pulmonary Medicine, Department of Internal Medicine, Taipei Medical University Hospital, Taipei, Taiwan; 5 Department of Medical Research, Taipei Medical University Hospital, Taipei, Taiwan; 6 Department of Medicine, University of Colorado School of Medicine, Aurora; 7 Graduate Institute of Data Science, College of Management, Taipei Medical University, Taipei, Taiwan

## Abstract

**Importance:**

Appropriate clinical decision-making relies on accurate data interpretation, which in turn relies on the use of suitable statistical models. Long tails and early crossover—2 features commonly observed in immune checkpoint inhibitor (ICI) survival curves—raise questions as to the suitability of Cox proportional hazards regression for ICI survival analysis. Cox proportional hazards–Taylor expansion adjustment for long-term survival data (Cox-TEL) adjustment may provide possible solutions in this setting.

**Objective:**

To estimate overall survival and progression-free survival benefits of ICI therapy vs chemotherapy using Cox-TEL adjustment.

**Data Sources:**

A PubMed search was performed for all cataloged publications through May 22, 2022.

**Study Selection:**

The search was restricted to randomized clinical trials with search terms for ICIs and lung cancer, melanoma, or urothelial carcinoma. The publications identified were further reviewed for inclusion.

**Data Extraction and Synthesis:**

Cox proportional hazards ratios (HRs) were transformed to Cox-TEL HRs for patients with short-term treatment response (ie, short-term survivor) (ST-HR) and difference in proportions for patients with long-term survival (LT-DP) by Cox-TEL. Meta-analyses were performed using a frequentist random-effects model.

**Main Outcomes and Measures:**

Outcomes of interest were pooled overall survival (primary outcome) and progression-free survival (secondary outcome) HRs, ST-HRs, and LT-DPs. Subgroup analyses stratified by cancer type also were performed.

**Results:**

A total of 1036 publications was identified. After 3 levels of review against inclusion criteria, 13 clinical trials (7 in non–small cell lung cancer, 3 in melanoma, and 3 in urothelial carcinoma) were selected for the meta-analysis. In the primary analysis, pooled findings were 0.75 (95% CI, 0.70-0.81) for HR, 0.86 (95% CI, 0.81-0.92) for ST-HR, and 0.08 (95% CI, 0.06-0.10) for LT-DP. In the secondary analysis, the pooled values for progression-free survival were 0.77 (95% CI, 0.64-0.91) for HR, 1.02 (95% CI, 0.84-1.24) for ST-HR, and 0.10 (95% CI, 0.06-0.14) for LT-DP.

**Conclusions and Relevance:**

This systematic review and meta-analysis of ICI clinical trial results noted consistently larger ST-HRs vs Cox HRs for ICI therapy, with an LT-DP of approximately 10%. These results suggest that Cox HRs may not provide a full picture of survival outcomes when the risk reduction from treatment is not constant, which may aid in the decision-making process of oncologists and patients.

## Key Points

**Question:**

Is there a difference in survival outcomes associated with immune checkpoint inhibitor therapy compared with chemotherapy when corrected for error introduced by Cox proportional hazards analysis?

**Findings:**

In this systematic review and meta-analysis of 13 clinical trials across 3 cancer types (non–small-cell lung cancer, urothelial carcinoma, and melanoma), the Cox proportional hazards–Taylor expansion adjustment for long-term survival data (Cox-TEL) adjustment method used to examine long-term survival probability noted an increment of approximately 10% over chemotherapy in patients with long-term survival who were receiving immune checkpoint inhibitor therapy.

**Meaning:**

The findings of this study suggest that Cox proportional hazard ratios may not provide a full picture of survival outcomes when the risk reduction from the treatment is not constant; Cox-TEL correction for appropriate data interpretation may be useful.

## Teaser

This systematic review and meta-analysis examines the use of adjusted Cox proportional hazards analysis for estimation of short- and long-term survival in patients with cancer treated with immune checkpoint inhibitors.

## Introduction

Early evidence of effective and durable immune response against cancer dates to the 1980s, when studies of interleukin-2 showed sustained response in approximately 10% of patients with advanced renal cell carcinoma and melanoma, with the unique hallmark of durable treatment effect: long tails in the Kaplan-Meier (KM) survival curve.^1,2^ This feature is now commonly observed in randomized clinical trials of immune checkpoint inhibitors (ICIs). Since the approval of the first ICI, ipilimumab, by the US Food and Drug Administration in 2011, ICIs have become part of standard therapy in cancer treatment.

Cox proportional hazards (PH) regression and the KM estimator are the standard methods used to compare survival benefits in oncology clinical trials. With long tails and early crossover in ICI survival curves, however, the PH assumption of the Cox model is violated, making Cox PH insufficient for data interpretation. Early crossover suggests poor response to ICI therapy in one subpopulation, while the long survival tail suggests durable response in another.

The PH cure model^3^ considers population survival as a mixture of patients without long-term survival (short-term survivors), with survival probabilities compared by HR, and patients in the long-tail segment of the survival curve (long-term survivors), with survival probabilities compared by difference in proportions (DP). Cox PH–Taylor expansion adjustment for long-term survival data (Cox-TEL) is a novel adjustment method developed based on the mathematical association between Cox PH and PH cure models. The Cox-TEL disassembles the study population into subgroups with and without long-term survival, providing the difference in proportions of survival probability for long-term survivors (LT-DP) and adjusted HR for short-term survivors (ST-HR).^4^ The only data required to perform the adjustment are Cox HR with 95% CIs and survival probabilities excerpted from KM curves, which are often made available in published studies.

**Figure 1.**
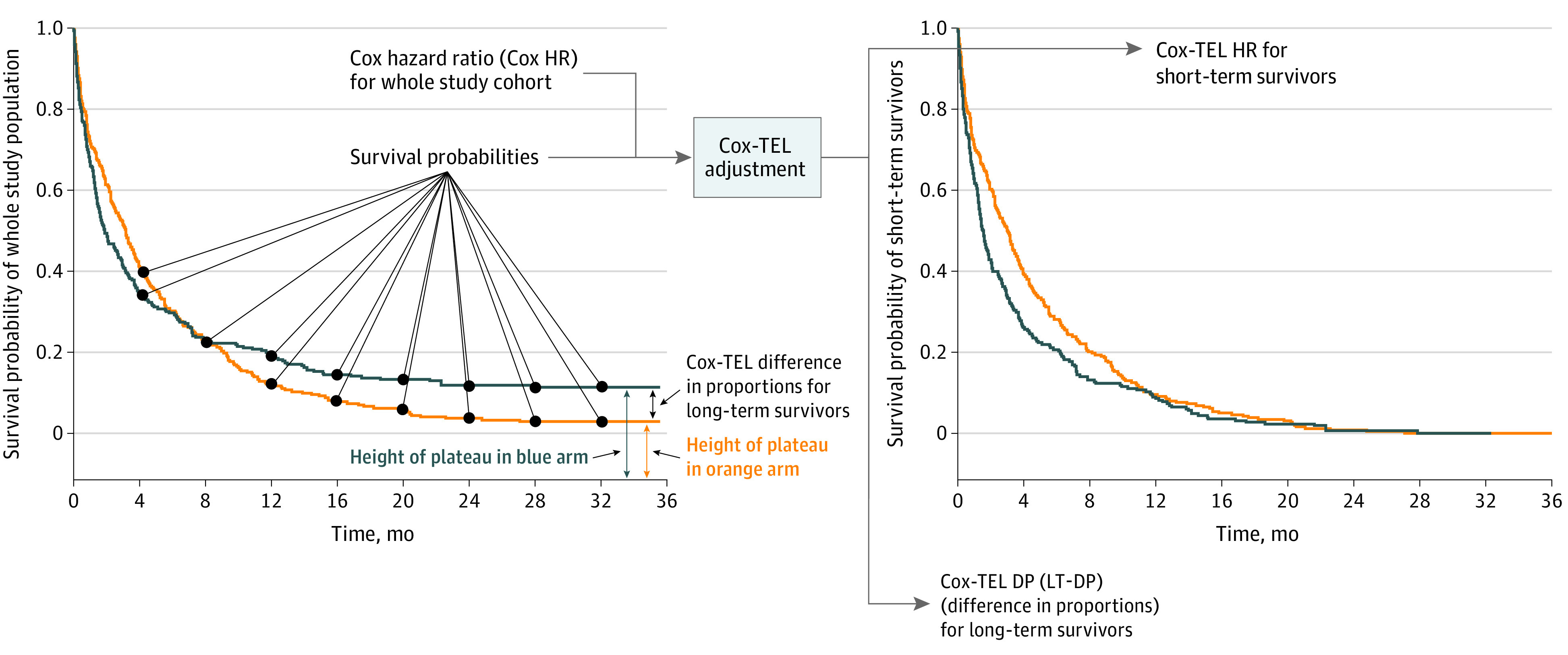
Cox Proportional Hazards–Taylor Expansion Adjustment for Long-term Survival Data (Cox-TEL) Adjustment Method Schema Cox hazard ratios (HRs) are transformed to Cox-TEL HRs (ST-HRs, for patients with short-term treatment response) and difference in proportions (LT-DPs, for responders with long-term survival) by Cox-TEL. The only data required to perform the adjustment are Cox HRs with 95% CIs and survival probabilities excerpted from Kaplan-Meier survival curves.

As illustrated in Figure 1 using the KEYNOTE-045 study as an example, Cox-TEL decomposes recaptured progression-free survival (PFS) KM curves into ST-HR and LT-DP, with the ST-HR curve showing a profile opposite that of the original KM curve.^5^ Additional examples of Cox-TEL adjustment are shown with recaptured overall survival (OS) and PFS KM curves for the CheckMate 017/057 studies (eMethods and eFigure 1 in the Supplement).^6^ In the context of the long-term survivor subpopulation, Cox-TEL adjustment corrects errors introduced by Cox PH analysis, which could otherwise lead to misinformed clinical decision-making.

Concerns with Cox HR analysis of data on long-term survival have been raised for nearly 2 decades, but alternatives are not yet widely accepted in the clinical trial community.^3,7,8,9^ With ICIs taking an increasingly central role in clinical oncology practice, however, the time has come to address this issue and provide a suitable statistical method to ensure better data interpretation and appropriate clinical decision-making for ICI therapy.

In this study, we examined the differences between HRs and ST-HRs and computed LT-DPs for 13 randomized clinical trials across 3 cancer types: non–small cell lung cancer (NSCLC), urothelial carcinoma (UC), and melanoma. Meta-analyses on these studies were performed, with OS the primary end point and PFS the secondary end point of ICI regimens.

## Methods

### Data Source and Selection Criteria

The PubMed database was searched for all cataloged publications through May 22, 2022. The search was restricted to randomized clinical trials as defined by the PubMed search engine. A total of 15 search terms were used. Each search term included the name of 1 ICI approved by the US Food and Drug Administration for treatment of NSCLC (nivolumab, pembrolizumab, cemiplimab, atezolizumab, durvalumab, and ipilimumab) plus lung cancer, for melanoma (nivolumab, pembrolizumab, atezolizumab, and ipilimumab) plus melanoma, or for UC (nivolumab, pembrolizumab, atezolizumab, avelumab, and ipilimumab) plus UC.

Publications identified were reviewed against 3 levels of predetermined inclusion and exclusion criteria. At level 1, publications were excluded if not phase 3 randomized clinical trials, not relevant to the selected cancer types, not comparing ICI treatment or ICI treatment plus chemotherapy (ICI regimen) vs chemotherapy, not reporting primary or secondary survival outcomes, or reporting trials in the neoadjuvant, adjuvant, or consolidation setting. At level 2, candidate publications were excluded for duplication or for not reporting OS results. At level 3, publications were excluded if (1) the study did not report HRs with 95% CIs, (2) the OS or PFS KM curves of the intention-to-treat (ITT) population did not meet piecewise regression criteria, (3) the study only included patients with programmed death-ligand 1 (PD-L1) expression greater than or equal to 50%, or (4) the publication reported interim results for a study with longer follow-up time available in an alternative source.

For studies not specifying an ITT population or if HRs with 95% CIs were not available for the specified ITT population, PD-L1 expression greater than or equal to 1% of the population was used as the ITT population.

The search and review for publication inclusion and exclusion were first done by a clinical reviewer (E.P.L.); studies that entered level 3 review were evaluated for final inclusion by a statistical reviewer (C.Y.H) assessing compliance with piecewise regression criteria. The findings are reported according to the Preferred Reporting Items for Systematic Reviews and Meta-analyses (PRISMA) guideline.^10^ The study was approved by the Vanderbilt University Medical Center Institutional Review Board according to principles of the Declaration of Helsinki.^11^

### Study Objectives and Outcomes

The objective of this study was to compare ICI treatment or ICI treatment plus chemotherapy (ICI regimen) vs chemotherapy-alone outcomes among the ITT population in patients with NSCLC, melanoma, and UC after Cox-TEL adjustment of reported Cox HRs. The primary end point of this study was OS, and the secondary end point was PFS. The outcomes were pooled OS Cox HR, Cox-TEL HR (ST-HR), and Cox-TEL difference in proportions (LT-DP). The ST-HR adjusts Cox HR for the study subpopulation identified as short-term survivors, and LT-DP expresses the additional fraction of the treated population with a response approximating cure. Subgroup analyses stratified by cancer type also were performed.

### Statistical Analysis

The Cox-TEL method was used to transform reported HRs for the ITT population in studies included for meta-analysis.^4^ The Cox-TEL method links the Cox PH model and PH cure models through their mathematic association and provides an algorithm to transform Cox HR to the more appropriate treatment-effect estimates as obtained from the PH cure model. The method requires as inputs only the reported Cox HR with 95% CI and KM curves. From these data, the Cox-TEL algorithm deconvolutes the 2 response subpopulations (short- and long-term survivors) and generates an appropriate output for each: more accurate HRs (ST-HR) for short-term survivors and, for long-term survivors, the incremental proportion of patients who achieve long-term survival approximating cure (LT-DP; eg, an LT-DP of 10% would indicate that, compared with the fraction of long-term survivors in the control group, that fraction, plus an additional 10% of the study population, achieved long-term survival in the treatment group).

Pairwise ST-HRs and LT-DPs along with the original HRs and the 95% CIs are reported. Frequentist random-effect meta-analysis was used to report pooled results. In the meta-analyses, the SEs of log(HR) and log(ST-HR) were calculated by converting 95% CIs using the following formula: SEs of log(HR) and log(ST-HR) = [log(upper bound of the CI) − log(lower bound of the CI)] / 3.92. The SEs of LT-DP were calculated by converting 95% CIs using the following formula: SE = (upper bound of the CI − lower bound of the CI) / 3.92. The Cochran *Q P* value ^12^ and the *I*^2^ statistic^13^ were used for heterogeneity testing. Publication bias was examined by the Egger and Begg-Mazumdar tests and was visualized using funnel plots.^14,15,16^ All data analyses were performed using R, version 3.6.1 and the R packages forestplot 1.10.1, netmeta 1.3-0, and meta 4.18-0.^17,18,19^

### Piecewise Regression Criteria

For each ICI trial, survival probabilities extracted from the KM survival curves at the prespecified time points were fitted to a piecewise regression with 2 knots for each arm. The knots were automatically selected by minimizing the sum of square errors between the predicted values and the extracted survival probabilities. Each of the fitted piecewise functions consisted of 3 line segments that constituted the 3 piecewise regression thresholds to determine whether an ICI study was eligible for meta-analysis. First, the slope of the last line segment should not depart from 0 as examined by the 95% CI of the estimated coefficient; if the 95% CI covered 0, the first threshold was met. Second, the relative slope change of the last line segment to the first line segment should be larger than 0.7. Third, the ratio of the length of the last line segment to the sum of the lengths of the first 2 line segments should be greater than 1/3. The study was included only if all 3 thresholds were met in the KM survival curves for both arms. The feasibility of these piecewise regression criteria has been tested and validated in 2 melanoma studies with median follow-up times of 6.9 and 5 years.^12,20^

## Results

### Publications and Studies

**Figure 2.**
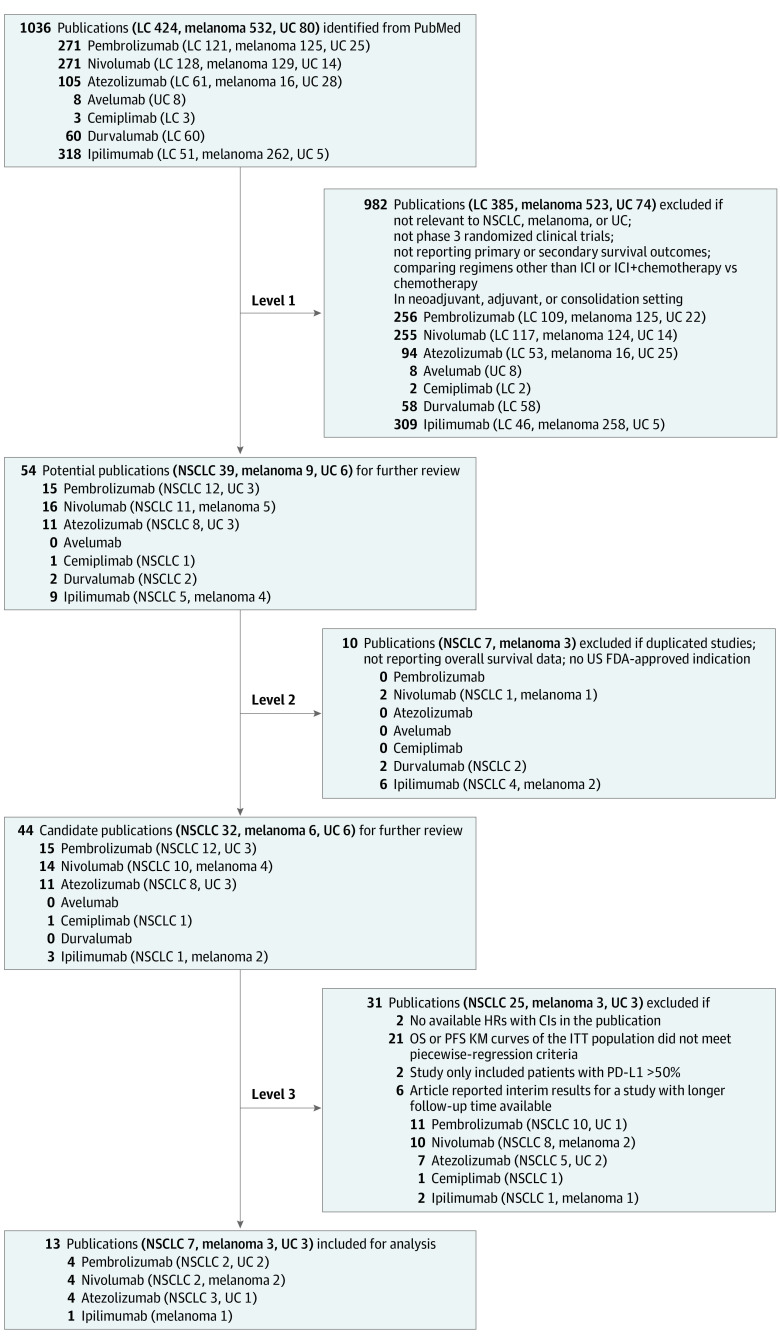
Study Selection Flowchart FDA indicates Food and Drug Administration; HRs, hazard ratios; ICI, immune checkpoint inhibitor; ITT, intention-to-treat; KM, Kaplan-Meier; LC, lung cancer; NSCLC, non–small cell lung cancer; OS, overall survival; PD-L1, programmed death-ligand 1; PFS, progression-free survival; and UC, urothelial carcinoma.

A total of 1036 publications was identified through the PubMed search. After level 1 review, 982 publications were excluded. Of the 54 publications remaining, 10 were excluded in level 2 review^12,21,22,23,24,25,26,27,28,29^ and 31 more were excluded^22,23,24,30,31,32,33,34,35,36,37,38,39,40,41,42,43,44,45,46,47,48,49,50,51,52,53,54,55,56,57^ in level 3 review (Table 1). A total of 13 publications was considered eligible for final analyses, including 7 for NSCLC (CheckMate 017/057, OAK, KEYNOTE-010, KEYNOTE-042, IMpower110, CheckMate 227, and IMpower132), 3 for melanoma (CA184-024, CheckMate 066, and CheckMate 037), and 3 for UC (KEYNOTE-045, IMvigor211, and KEYNOTE-361) (Figure 2 and Table 2).^5,6,12,21,58,59,60,61,62,63,64,65,66^

**Table 1.** Studies and Publications for Level 3 Review, After Exclusions at Levels 1 and 2

Phase 3 trials screened	Trials included	Median follow-up, ≥24 mo	PR criteria^a^	Publications included	Source
**Non–small cell lung cancer**					
CheckMate 017/057	Yes	No	FT	No	Brahmer et al,^30^ 2015
		No	FF	No	Borghaei et al,^31^ 2015
		No	0	No	Horn et al,^32^ 2017
		Yes	TT	No	Vokes et al,^33^ 2018
		Yes	TT	Yes	Borghaei et al,^6^ 2021
OAK	Yes	No	FF	No	Rittmeyer et al,^34^ 2017
		Yes	TT	No	Fehrenbacher et al,^35^ 2018
		Yes	TT	Yes	Mazieres et al,^58^ 2021
KEYNOTE-010	Yes	No	FF	No	Herbst et al,^36^ 2016
		No	TT	No	Herbst et al,^37^ 2020
		Yes	TT	Yes	Herbst et al,^59^ 2021
KEYNOTE-042	Yes	No	TT	Yes	Mok et al,^60^ 2019
IMpower110	Yes	No	FT	No	Herbst et al,^38^ 2020
		Yes	TT	Yes	Jassem et al,^61^ 2021
CheckMate 227	Yes	No	0	No	Hellmann et al,^39^ 2018
		Yes	TT	Yes	Hellmann et al,^21^ 2019
IMpower132	Yes	Yes	TT	Yes	Nishio et al,^62^ 2021
KEYNOTE-024	No	No	FT	No	Reck et al,^40^ 2016
		No	FT	No	Reck et al,^41^ 2019
		Yes	TT	No	Reck et al,^42^ 2021
KEYNOTE-189	No	No	FF	No	Gandhi et al,^43^ 2018
		No	FT	No	Gadgeel et al,^44^ 2020
		Yes	FT	No	Rodríguez-Abreu et al,^45^ 2021
KEYNOTE-407	No	No	FF	No	Paz-Ares et al,^46^ 2018
		No	FF	No	Paz-Ares et al,^47^ 2020
IMpower130	No	No	FF	No	West et al,^48^ 2019
IMpower131	No	No	FF	No	Jotte et al,^49^ 2020
CheckMate 026	No	No	FF	No	Carbone et al,^50^ 2017
CheckMate 9LA	No	No	FF	No	Paz-Ares et al,^22^ 2021
		Yes	FT	No	Reck et al,^23^ 2021
EMPOWER-Lung 1	No	No	FF	No	Sezer et al,^51^ 2021
NCT01285609	No	Yes	FF	No	Govindan,^52^ 2017
**Melanoma**					
CA184-024	Yes	Yes	TT	No	Robert et al,^24^ 2011
		Yes	TT	Yes	Maio et al,^12^ 2015
CheckMate 066	Yes	No	FF	No	Robert et al,^53^ 2015
		Yes	TT	No	Ascierto et al,^54^ 2019
		Yes	TT	Yes	Robert et al,^63^ 2020
CheckMate 037	Yes	Yes	TT	Yes	Larkin et al,^64^ 2018
**Urothelial cancer**					
KEYNOTE-045	Yes	No	FF	No	Bellmunt et al,^55^ 2017
		Yes	TT	Yes	Fradet et al,^5^ 2019
IMvigor211	Yes	No	FF	No	Powles et al,^56^ 2018
		Yes	TT	Yes	van der Heijden et al,^65^ 2021
KEYNOTE-361	Yes	Yes	TT	Yes	Powles et al,^66^ 2021
IMvigor130	No	No	FF	No	Galsky et al,^57^ 2020

Abbreviations: Cox-TEL, Cox proportional hazards–Taylor expansion adjustment for long-term survival data; PR, piecewise regression; TT, OS KM curve for intention-to-treat population met criteria for Cox-TEL adjustment.

^a^ PR criteria: annotation indicates whether experimental and control arms met (T, TRUE) or did not meet (F, FALSE) all 3 piecewise regression criteria. The first TRUE/FALSE indicator is for the experimental arm, and the second, for the control arm. For example, FT would indicate: experimental arm did not meet criteria; control arm met criteria.

**Table 2.** Phase 3 Trials Included in the Meta-analysis^a^

Phase 3 trials included	Median follow-up ≥24 mo	Source
**Non–small cell lung cancer**		
CheckMate 017/057	Yes	Borghaei et al,^6^ 2021
OAK	Yes	Mazieres et al,^58^ 2021
KEYNOTE-010	Yes	Herbst et al,^59^ 2021
KEYNOTE-042	No	Mok et al,^60^ 2019
IMpower110	Yes	Jassem et al,^61^ 2021
CheckMate 227	Yes	Hellmann et al,^21^ 2019
IMpower132	Yes	Nishio et al,^62^ 2021
**Melanoma**		
CA184-024	Yes	Maio et al,^12^ 2015
CheckMate 066	Yes	Robert et al,^63^ 2020
CheckMate 037	Yes	Larkin et al,^64^ 2018
**Urothelial carcinoma**		
KEYNOTE-045	Yes	Fradet et al,^5^ 2019
IMvigor211	Yes	van der Heijden et al,^65^ 2021
KEYNOTE-361	Yes	Powles et al,^66^ 2021

^a^ The overall survival Kaplan-Meier curves for the intention-to-treat population in all trials met the piecewise regression criteria.

The PD-L1 greater than or equal to 1% population was designated as the ITT population for KEYNOTE-010, KEYNOTE-042, and IMpower110 because an ITT population was not specified, and in CheckMate 227 because HRs with 95% CIs were not available for the specified ITT population. Heterogeneity test results are reported in the eTable in the Supplement, and publication bias results are shown in eFigure 2 in the Supplement.

### Primary Outcomes

For NSCLC, the ST-HRs for OS were larger than the Cox HRs. In all 4 first-line ICI studies (CheckMate 227, KEYNOTE-042, IMpower110, and IMpower132), the ST-HRs were statistically nonsignificant but were suggestive of benefit in the 3 second-line ICI regimen studies: CheckMate 017/057 (0.85; 95% CI, 0.74-0.98), OAK (0.84; 95% CI, 0.74-0.96), and KEYNOTE-010 (0.83; 95% CI, 0.72-0.95). The LT-DP for OS was greatest in CheckMate 227 (0.11; 95% CI, 0.01-0.21), which used ICI combination therapy, and statistically nonsignificant in IMpower110, IMpower132, and OAK. Calculated LT-DPs were similar in CheckMate 017/057 (0.09; 95% CI, 0.05-0.14), KEYNOTE-010 (0.08; 95% CI, 0.03-0.13), and KEYNOTE-042 (0.09; 95% CI, 0.01-0.16).

For UC, the ST-HRs for OS also were larger than the Cox HRs. In IMvigor211, the ST-HR was statistically nonsignificant, but the findings remained suggestive of benefit in KEYNOTE-045 (0.77; 95% CI, 0.63-0.94). The LT-DPs were similar in both studies: 0.09 (95% CI, 0.01-0.19) for KEYNOTE-045 and 0.08 (95% CI, 0.02-0.15) for IMvigor211.

For melanoma, the ST-HRs for OS were once again larger than the Cox HRs. In CA184-024 and CheckMate 037, ST-HRs were statistically nonsignificant, but the findings remained suggestive of benefit in CheckMate 066 (0.62; 95% CI, 0.49-0.78). The LT-DP was greatest in CheckMate 066 (0.20; 95% CI, 0.09-0.30), followed by CA184-024 (0.09; 95% CI, 0.02-0.16), and was statistically nonsignificant in CheckMate 037.

**Figure 3.**
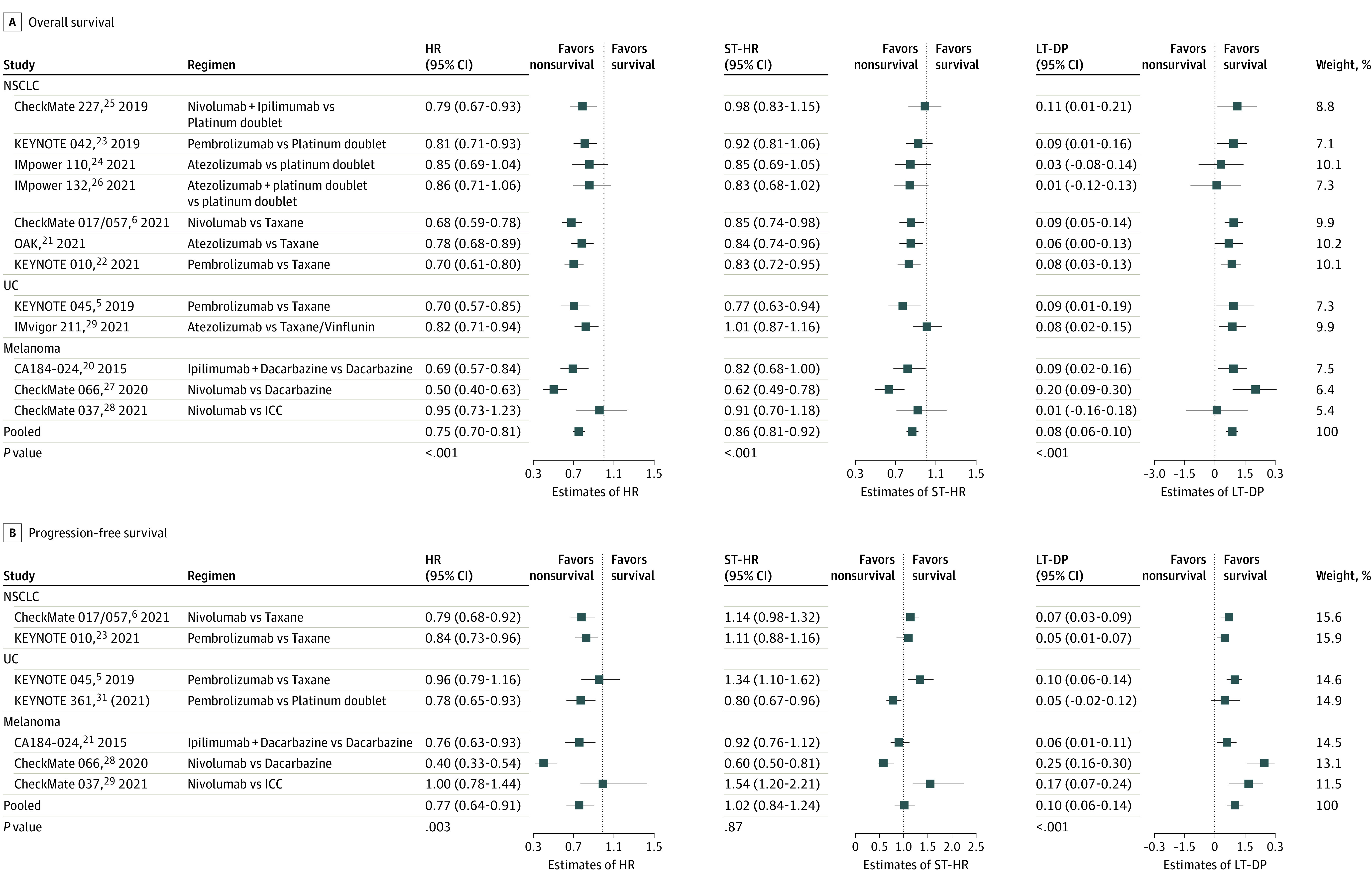
Association of Immune Checkpoint Inhibitors With Overall Survival and Progression-Free Survival Cox hazard ratios (HRs), Cox-Taylor expansion adjustment for short-term survival data (Cox-TEL) HRs (ST-HR), and difference in proportions of survival probability for long-term survivors (LT-DP) illustrate survival end points of included studies before and after Cox-TEL adjustment for overall survival (A) and progression-free survival (B). Pooled end points are meta-analysis results. The weight for each study is inversely proportional to the within-study variance of log(HR) plus the between-studies variance. NSCLC indicates non–small cell lung cancer; UC, urothelial carcinoma.

In all 3 cancer types, the ST-HR for OS was consistently larger than the Cox HR, suggesting the contribution of the long-term survivor population to the estimation of Cox HR. The pooled findings for OS were 0.75 (95% CI, 0.70-0.81) for HR, 0.86 (95% CI, 0.81-0.92) for ST-HR, and 0.08 (95% CI, 0.06-0.10) for LT-DP (Figure 3A).

### Secondary Outcomes

As observed in the OS data, the ST-HRs for PFS remained consistently larger than the Cox HRs, suggesting the contribution of long-term survivors to the estimation of HRs in PFS. The ST-HRs were greater than 1, suggesting risks with ICI regimen use for short-term disease control, compared with chemotherapy, in CheckMate 017/057 (1.14; 95% CI, 0.98-1.32) and KEYNOTE-010 (1.11; 95% CI, 0.88-1.16) for NSCLC, in KEYNOTE-045 (1.34; 95% CI, 1.10-1.62) for UC, and in CheckMate 037 for melanoma (1.54; 95% CI, 1.20-2.21). The ST-HRs were statistically nonsignificant in CA184-024 but remained significant in KEYNOTE-361 (0.80; 95% CI, 0.67-0.96) and CheckMate 066 (0.60; 95% CI, 0.50-0.81). The LT-DPs were statistically significant in all the studies except KEYNOTE-361. The pooled findings for PFS were 0.77 (95% CI, 0.64-0.91) for HR, 1.02 (95% CI, 0.84-1.24) for ST-HR, and 0.10 (95% CI, 0.06-0.14) for LT-DP (Figure 3B).

#### OS Benefit Stratified by Cancer Type

With meta-analysis stratified by cancer type, similar patterns emerged. The pooled LT-DP for melanoma (0.11; 95% CI, 0.01-0.20) was greater than that for NSCLC (0.08; 95% CI, 0.05-0.10) and UC (0.08; 95% CI, 0.03-0.14). Conversely, the pooled HR for melanoma (0.69; 95% CI, 0.49-0.96) was smaller than those for NSCLC (0.77; 95% CI, 0.72-0.82) and UC (0.77; 95% CI, 0.66-0.90). Pooled ST-HRs remained larger than pooled Cox HRs: 0.78 (95% CI, 0.62-0.97) for melanoma, 0.87 (95% CI, 0.82-0.92) for NSCLC, and 0.89 (95% CI, 0.68-1.16) for UC (eFigure 3 in the Supplement).

#### PFS Benefit Stratified by Cancer Type

The pooled ST-HR for PFS indicated risks with ICI regimen use for NSCLC (1.12; 95% CI, 1.02-1.24) and UC (1.03; 95% CI, 0.62-1.71) and was statistically nonsignificant for melanoma (0.94; 95% CI, 0.58-1.51). The pooled LT-DP was 0.06 (95% CI, 0.04-0.08) for NSCLC, 0.08 (95% CI, 0.04-013) for UC, and 0.16 (95% CI, 0.04-0.28) for melanoma (eFigure 4 in the Supplement).

## Discussion

To our knowledge, this study represents the first comprehensive revisit of randomized clinical trial results on use of ICI therapy in NSCLC, UC, and melanoma, reporting survival end points before and after Cox-TEL adjustment in 13 ICI randomized clinical trials across 3 cancer types. Meta-analyses suggest consistently larger ST-HRs than Cox HRs for patients with short-term survival who are receiving ICI therapy and an approximate 10% survival probability increment (LT-DP) for those with long-term survival. In survival data with treatment effect not constant over time, Cox HRs cannot provide a full picture of survival outcomes; however, the Cox-TEL adjustment can better interpret such survival data. This finding is especially useful for oncologists because ICIs now represent a mainstay of cancer therapy.

In the primary analyses, we noted a pooled Cox HR for OS of 0.75—in line with prior ICI meta-analyses and consistent with the current understanding of survival benefit for approximately 20% to 40% of patients who receive ICI therapy.^67,68^ With Cox-TEL deconvolution of patient subpopulations based on ICI treatment response, however, the pooled ST-HR was calculated as 0.86 and the pooled LT-DP as 0.08.

In the secondary analyses, the pooled Cox HR for PFS was 0.77, similar to prior estimations.^68^ The pooled ST-HR, however, was 1.02—a signal to the oncologist suggesting possible harm with use of ICI therapy for disease control. In contrast, the pooled LT-DP for PFS was 0.10, indicating a 10% increment in long-term PFS probability for long-term survivors, compared with chemotherapy.

Although crossover is not typical in PFS data, OS data almost always show crossover, either within the study period or off-study. Therefore, the 10% long-term survival probability increment estimated from PFS data may be more accurate, with the 8% estimated from OS data an underestimation. Taken together, these data suggest an approximately 10% long-term survival benefit for individuals with long-term survival who are receiving ICI therapy vs those receiving chemotherapy.

In subgroup analysis, the pooled LT-DP for OS was larger in patients with melanoma than in NSCLC or UC. This finding, consistent with earlier observations of durable ICI therapy benefit in a relatively high proportion of patients with melanoma,^69^ further supports the reliability of the Cox-TEL adjustment method.

In the ICI clinical research field, many unresolved issues remain, for example, the association of PD-L1 expression level with long-term ICI therapy survival benefits, differences in long-term survival in the mono-ICI vs dual-ICI therapy setting, and appropriate follow-up duration for the first report of study outcomes. Further research is needed to address these issues.

### Limitations

This study has limitations. A major limitation is the small number of randomized clinical trials on ICI treatment with sufficient follow-up. Only 2 ICI therapy plus chemotherapy combination trials met the inclusion criteria for Cox-TEL adjustment, limiting conclusions regarding combination therapy. In addition, the Cox-TEL adjustment method uses reported Cox HRs and survival probabilities extracted from reported KM survival curves. Although robust and practical, this method is necessarily limited in adjusting processed data, and more informative conclusions could be drawn with direct analysis of the raw data under a cure model.

## Conclusions

To our knowledge, this study is the first to revisit published ICI therapy trial results with correction for error introduced by Cox PH analysis and provides a clearer picture of ICI treatment effect. For patients receiving ICI therapy who are short-term survivors with ICI treatment of cancer, ST-HRs appear to be consistently larger than Cox HRs. For patients receiving ICI therapy who are long-term survivors, the Cox-TEL adjustment method estimates a long-term survival probability increment of approximately 10%, compared with chemotherapy. These results are of particular importance for evidence-based clinical decision-making in oncology practice, where ICI treatment has become a mainstay of medical therapy.

## Notes

Supplement. **eMethods.** Simulations to Recapture Kaplan-Meier Survival Curves of KEYNOTE-045 and CheckMate 017/057 **eFigure 1.** Recaptured Kaplan-Meier Curves for KEYNOTE-045 and CheckMate 017/057 by Simulation **eFigure 2.** Funnel Plots for Publication Bias of Meta-analyses **eFigure 3.** Subgroup Analyses of Overall Survival **eFigure 4.** Subgroup Analyses of Progression-Free Survival **eTable.** Results of Heterogeneity Tests for Meta-analyses
