# Supplementary material for: Analysis of Cancer Survival Associated With Immune Checkpoint Inhibitors After Statistical Adjustment: A Systematic Review and Meta-analyses
Source: JAMA Netw Open. 2022 Aug 17;5(8):e2227211. doi: 10.1001/jamanetworkopen.2022.27211 (PMC9386543; doi:10.1001/jamanetworkopen.2022.27211)
Supplement: Supplement. — eMethods. Simulations to Recapture Kaplan-Meier Survival Curves of KEYNOTE-045 and CheckMate 017/057 eFigure 1. Recaptured Kaplan-Meier Curves for KEYNOTE-045 and CheckMate 017/057 by Simulation eFigure 2. Funnel Plots for Publication Bias of Meta-analyses eFigure 3. Subgroup Analyses of Overall Survival eFigure 4. Subgroup Analyses of Progression-Free Survival eTable. Results of Heterogeneity Tests for Meta-analyses [file jamanetwopen-e2227211-s001.pdf]

Supplementary Online Content

Lin EPY, Hsu CY, Berry L, Bunn P, Shyr Y. Analysis of cancer survival associated with immune checkpoint inhibitors after statistical adjustment: a systematic review and meta-analyses. *JAMA Netw Open*. 2022;5(8):e2227211. doi:10.1001/jamanetworkopen.2022.27211

eMethods. Simulations to Recapture Kaplan-Meier Survival Curves of KEYNOTE-045 and CheckMate 017/057

eFigure 1. Recaptured Kaplan-Meier Curves for KEYNOTE-045 and CheckMate 017/057 by Simulation

eFigure 2. Funnel Plots for Publication Bias of Meta-analyses

eFigure 3. Subgroup Analyses of Overall Survival

eFigure 4. Subgroup Analyses of Progression-Free Survival

eTable. Results of Heterogeneity Tests for Meta-analyses

This supplementary material has been provided by the authors to give readers additional information about their work.

© 2022 Lin EPY et al. *JAMA Network Open.*

eMethod: Simulations to recapture Kaplan-Meier survival curves of KEYNOTE 045 and CheckMate 017/057

eFigure 1 (A) and (B) show survival curves generated from simulated data based on KEYNOTE 045 of chemotherapy ($n_0 = 272$) versus pembrolizumab ($n_1 = 270$) for OS or PFS of patients with advanced UC (1). OS or PFS probabilities at 4-month intervals were extracted from the KM survival curves (Reference 1, Figure 1A-B), and the proportions of long-term responders were assumed to be 0.13 (0.01) and 0.22 (0.11) in the chemotherapy and pembrolizumab arms, respectively. We fitted mixture cure models with Weibull distributions to the extracted probabilities by the nonlinear least-squares method, and we obtained two Weibull distributions with a common shape parameter of 1.133 (0.690) and different scale parameters of 0.697 (0.312) and 0.789 (0.235) for the two arms, respectively. Accordingly, event times were generated from mixture cure models with the estimated Weibull distributions and the proportions of long-term responders. Moreover, we assumed the same uniform distribution Uniform(2, 3) of censoring time for both arms. Then, we obtained the simulated data and produced the unadjusted KM curves as shown in eFigure 1A-B.

Similarly, eFigure 1 (C) and (D) are simulations from the CheckMate 017/057 trial of docetaxel ($n_0 = 427$) versus nivolumab ($n_1 = 427$) for OS or PFS of patients with advanced NSCLC (2). OS or PFS probabilities at 6-month intervals were extracted from the KM survival curves (Reference 2, Figure 1A for OS and Figure 2A for PFS), and the proportions of long-term responders were assumed to be 0.02 (0.00) and 0.11 (0.07) in the docetaxel and nivolumab arms, respectively. Following the same procedure described as above, we obtained two Weibull distributions with a common shape parameter of 0.939 (0.638) and different scale parameters of 0.931 (0.308) and 1.120 (0.350) for the two arms, respectively. Also, we assumed the same uniform distribution Uniform(5, 6.5) of censoring time for both arms. Thus, we obtained the simulated data and the unadjusted KM curves as shown in eFigure 1C-D.

© 2022 Lin EPY et al. *JAMA Network Open.*

eReferences

1. Fradet Y, Bellmunt J, Vaughn DJ, et al. Randomized phase III KEYNOTE-045 trial of pembrolizumab versus paclitaxel, docetaxel, or vinflunine in recurrent advanced urothelial cancer: results of >2 years of follow-up. Ann Oncol. 2019;30(6):970-976.
2. Borghaei H, Gettinger S, Vokes EE, et al. Five-Year Outcomes From the Randomized, Phase III Trials CheckMate 017 and 057: Nivolumab Versus Docetaxel in Previously Treated Non-Small-Cell Lung Cancer. J Clin Oncol. 2021;39(7):723-733.

© 2022 Lin EPY et al. *JAMA Network Open.*

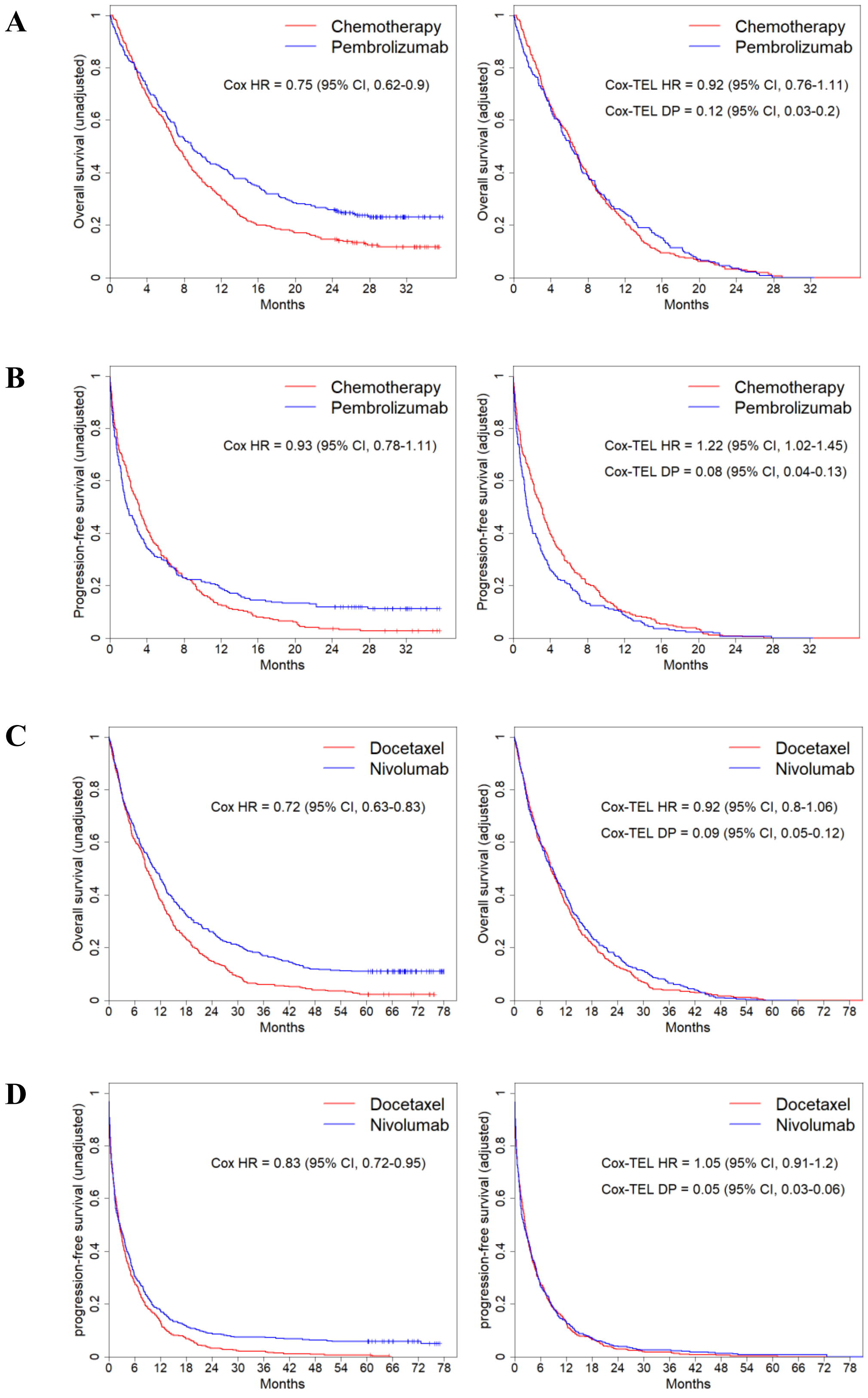

eFigure 1. Recaptured Kaplan-Meier curves for KEYNOTE 045 and CheckMate 017/057 by simulation. KEYNOTE 045 unadjusted and adjusted **(A)** OS and **(B)** PFS curves. CheckMate 017/057 unadjusted and adjusted **(C)** OS and **(D)** PFS curves. Cox HRs, Cox-TEL HRs and Cox-TEL DPs are shown.

© 2022 Lin EPY et al. *JAMA Network Open.*

HR | **ST-HR** | **LT-DP**

Fig3

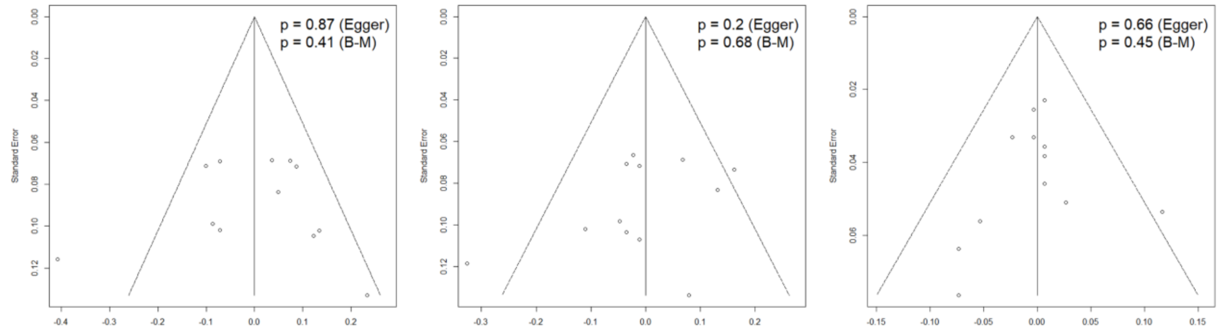

Fig4

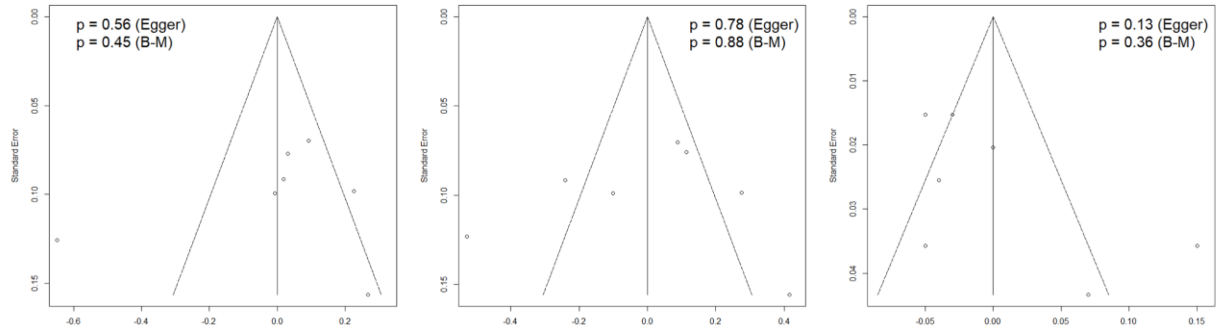

eFig3A

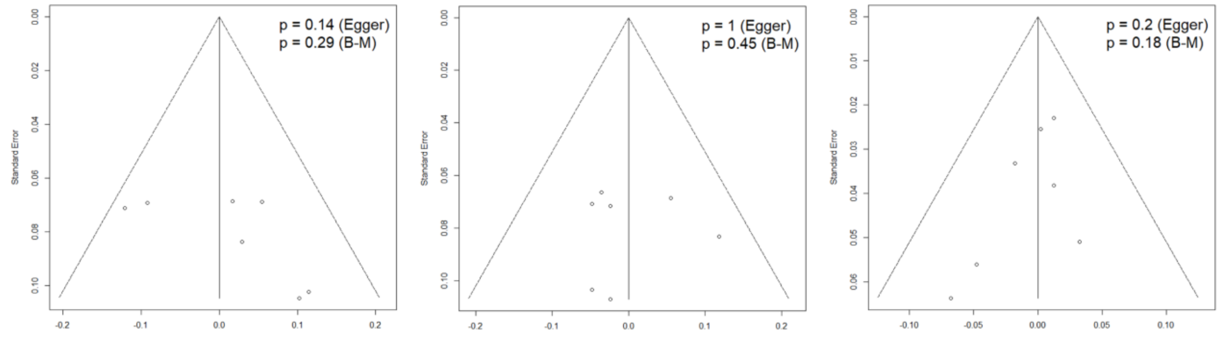

eFig3B

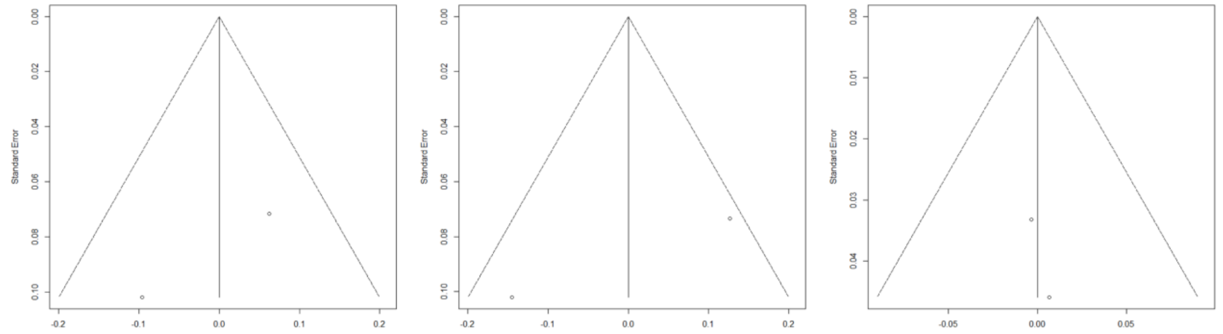

eFig3C

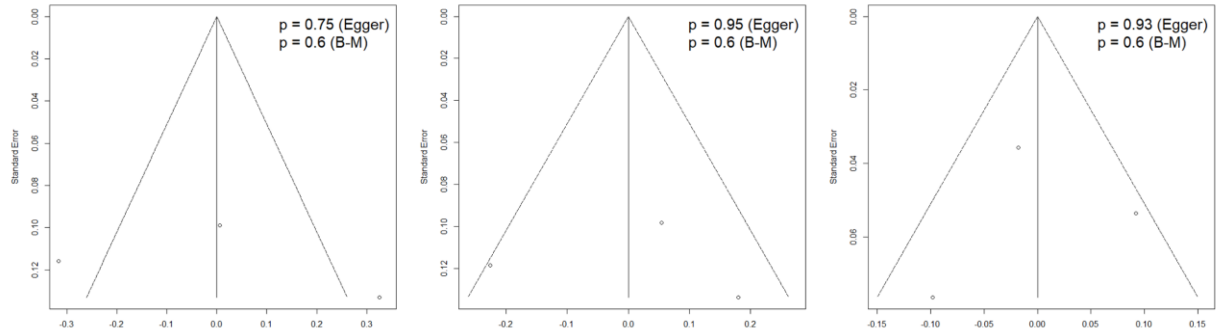

© 2022 Lin EPY et al. *JAMA Network Open.*

eFig4A

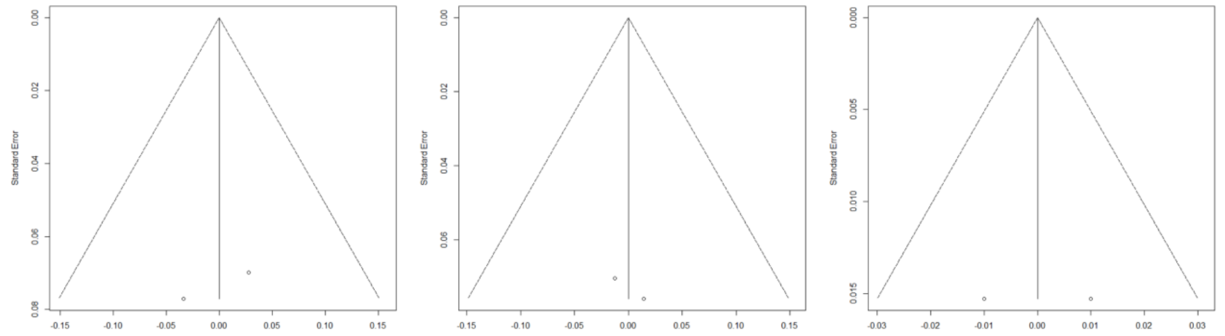

eFig4B

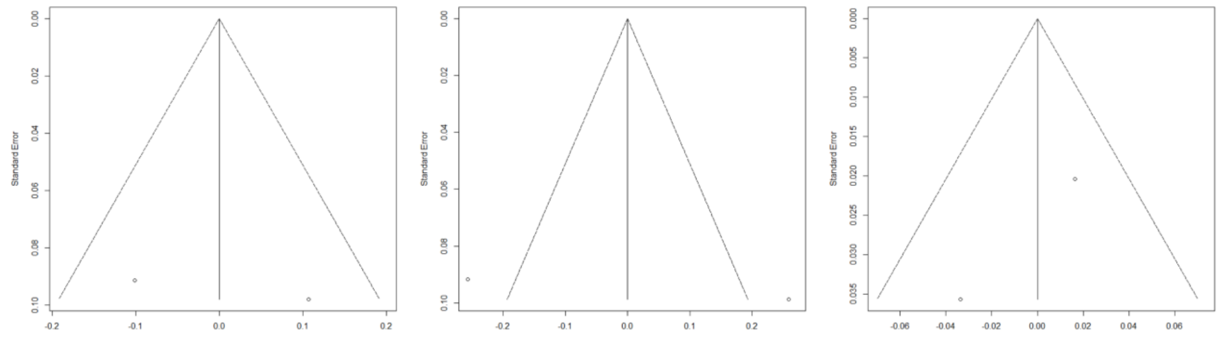

eFig4C

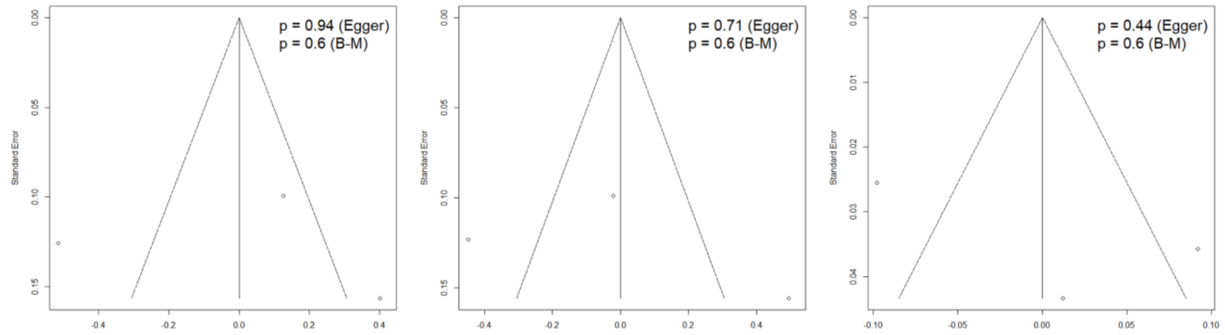

eFigure 2. Funnel plots for publication bias of meta-analyses. p = P-value that were obtained from Egger test (Egger) and Begger-Mazumdar test (B-M). The p-values for meta-analysis using only two studies cannot be calculated.

© 2022 Lin EPY et al. *JAMA Network Open.*

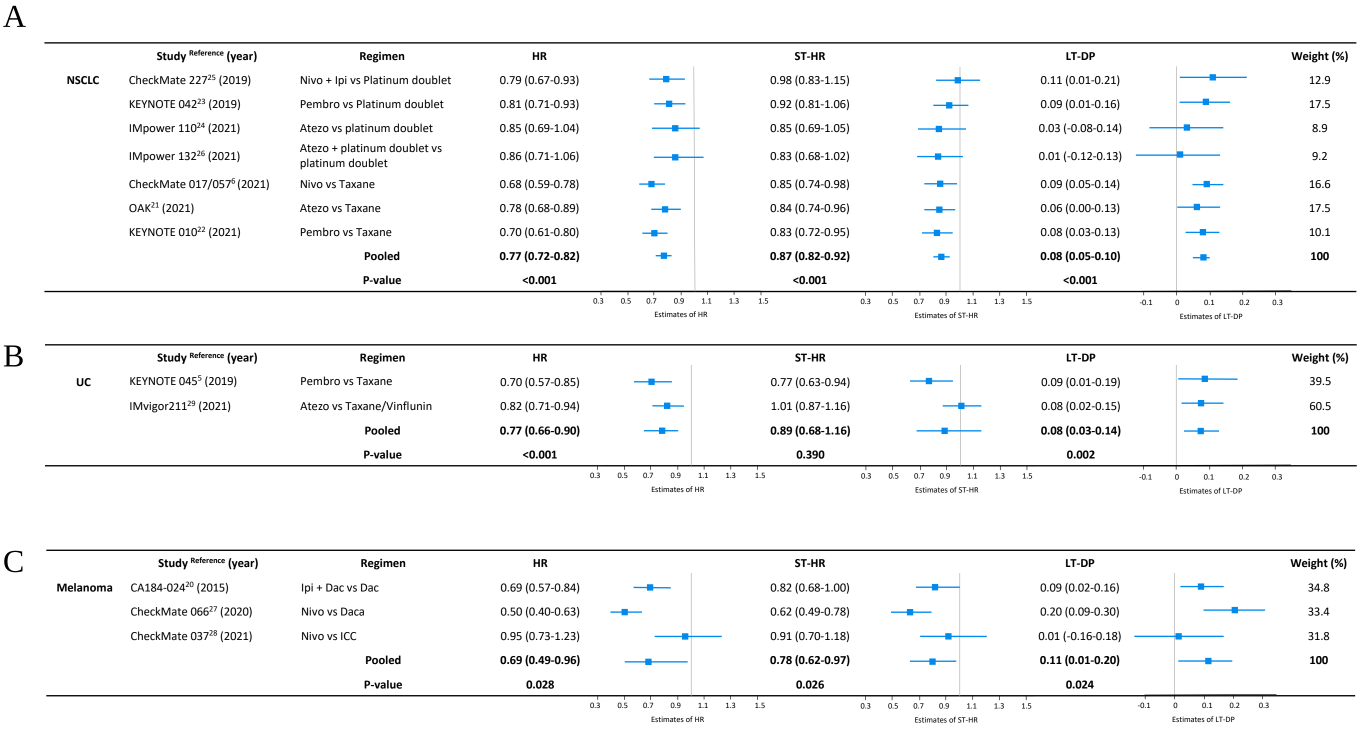

A

	Study [Reference] (year)	Regimen	HR	ST-HR	LT-DP	Weight (%)
**NSCLC**	CheckMate 227[25] (2019)	Nivo + Ipi vs Platinum doublet	0.79 (0.67-0.93)	0.98 (0.83-1.15)	0.11 (0.01-0.21)	12.9
	KEYNOTE 042[23] (2019)	Pembro vs Platinum doublet	0.81 (0.71-0.93)	0.92 (0.81-1.06)	0.09 (0.01-0.16)	17.5
	IMpower 110[24] (2021)	Atezo vs platinum doublet	0.85 (0.69-1.04)	0.85 (0.69-1.05)	0.03 (-0.08-0.14)	8.9
	IMpower 132[26] (2021)	Atezo + platinum doublet vs platinum doublet	0.86 (0.71-1.06)	0.83 (0.68-1.02)	0.01 (-0.12-0.13)	9.2
	CheckMate 017/057[6] (2021)	Nivo vs Taxane	0.68 (0.59-0.78)	0.85 (0.74-0.98)	0.09 (0.05-0.14)	16.6
	OAK[21] (2021)	Atezo vs Taxane	0.78 (0.68-0.89)	0.84 (0.74-0.96)	0.06 (0.00-0.13)	17.5
	KEYNOTE 010[22] (2021)	Pembro vs Taxane	0.70 (0.61-0.80)	0.83 (0.72-0.95)	0.08 (0.03-0.13)	10.1
		**Pooled**	**0.77 (0.72-0.82)**	**0.87 (0.82-0.92)**	**0.08 (0.05-0.10)**	**100**
		**P-value**	**<0.001**	**<0.001**	**<0.001**	

B

	Study [Reference] (year)	Regimen	HR	ST-HR	LT-DP	Weight (%)
**UC**	KEYNOTE 045[5] (2019)	Pembro vs Taxane	0.70 (0.57-0.85)	0.77 (0.63-0.94)	0.09 (0.01-0.19)	39.5
	IMvigor211[29] (2021)	Atezo vs Taxane/Vinflunin	0.82 (0.71-0.94)	1.01 (0.87-1.16)	0.08 (0.02-0.15)	60.5
		**Pooled**	**0.77 (0.66-0.90)**	**0.89 (0.68-1.16)**	**0.08 (0.03-0.14)**	**100**
		**P-value**	**<0.001**	**0.390**	**0.002**	

C

	Study [Reference] (year)	Regimen	HR	ST-HR	LT-DP	Weight (%)
**Melanoma**	CA184-024[20] (2015)	Ipi + Dac vs Dac	0.69 (0.57-0.84)	0.82 (0.68-1.00)	0.09 (0.02-0.16)	34.8
	CheckMate 066[27] (2020)	Nivo vs Daca	0.50 (0.40-0.63)	0.62 (0.49-0.78)	0.20 (0.09-0.30)	33.4
	CheckMate 037[28] (2021)	Nivo vs ICC	0.95 (0.73-1.23)	0.91 (0.70-1.18)	0.01 (-0.16-0.18)	31.8
		**Pooled**	**0.69 (0.49-0.96)**	**0.78 (0.62-0.97)**	**0.11 (0.01-0.20)**	**100**
		**P-value**	**0.028**	**0.026**	**0.024**	

eFigure 3. Subgroup analyses of overall survival. (A) NSCLC, **(B)** UC, **(C)** melanoma. Forest plots of Cox HRs (HR), Cox-TEL HRs (ST-HR), and LT-DPs illustrate overall survival endpoints of included studies before and after Cox-TEL adjustment. Individual and pooled HRs, ST-HRs, and LT-DPs are shown with 95% CI. Pooled endpoints are meta-analysis results. The weight for each study is inversely proportional to the within-study variance of log(HR) plus the between-studies variance. Abbreviations: Nivo: nivolumab; Pembro: pembrolizumab; Atezo: atezolizumab; Ipi: ipilimumab; Dac: dacarbazine; ICC: investigator’s choice chemotherapy (dacarbazine or carboplatin plus paclitaxel).

© 2022 Lin EPY et al. *JAMA Network Open.*

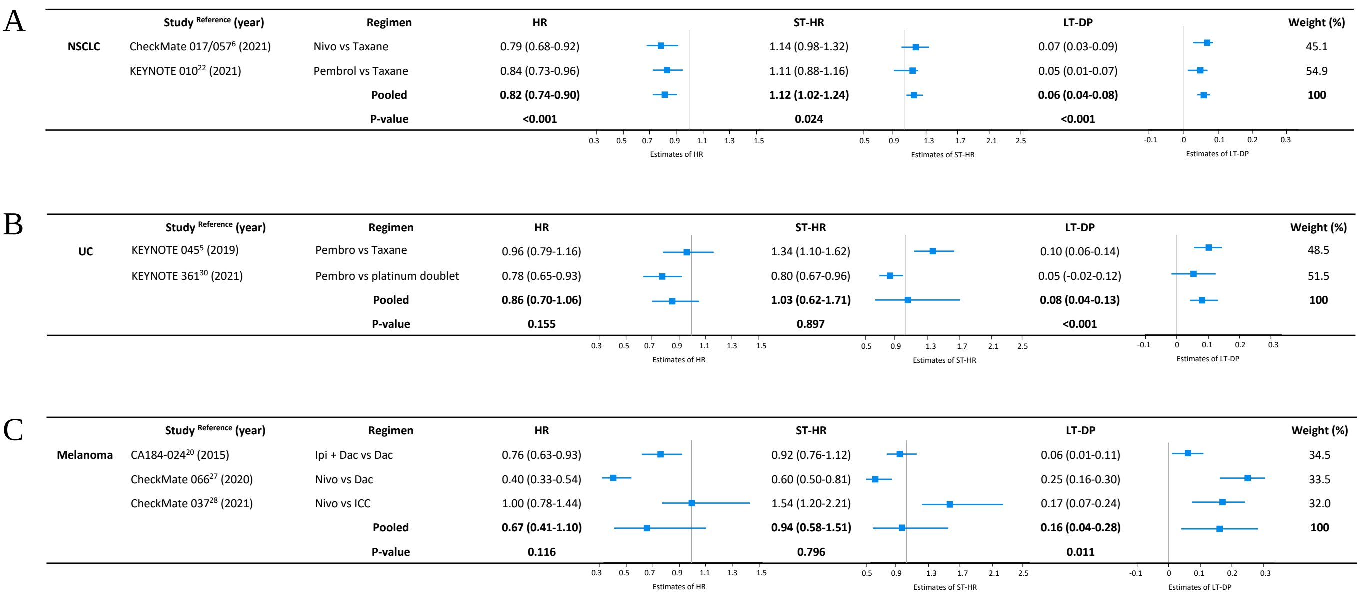

eFigure 4. Subgroup analyses of progression-free survival. (A) NSCLC and **(B)** melanoma. Forest plots of Cox HRs (HR), Cox-TEL HRs (ST-HR), and LT-DPs illustrate progression-free survival endpoints of included studies before and after Cox-TEL adjustment. Individual and pooled HRs, ST-HRs, and LT-DPs are shown with 95% CI. Pooled endpoints are meta-analysis results. The weight for each study is inversely proportional to the within-study variance of log(HR) plus the between-studies variance. Abbreviations: Nivo: nivolumab; Pembro: pembrolizumab; Atezo: atezolizumab; Ipi: ipilimumab; Dac: dacarbazine; ICC: investigator's choice chemotherapy (dacarbazine or carboplatin plus paclitaxel).

© 2022 Lin EPY et al. *JAMA Network Open.*

eTable 1. Results of heterogeneity tests for meta-analyses.

OS	HR		ST-HR		LT-DP	
	CQ-P	I^2	CQ-P	I^2	CQ-P	I^2
**Figure 3 OS**	0.01	58% (95% CI 20%-78%)	0.08	39% (95% CI 0%-69%)	0.64	0% (95% CI 0%-48%)
**Figure 4 PFS**	<0.01	84% (95% CI 68%-92%)	<0.01	87% (95% CI 75%-93%)	<0.01	82% (95% CI 64%-91%)
**eFigure 3A**	0.27	0% (95% CI 0%-65%)	0.70	0% (95% CI 0%-54%)	0.82	0% (95% CI 0%-40%)
**eFigure 3B**	0.20	-	0.03	-	0.86	-
**eFigure 3C**	<0.01	85% (95% CI 56%-95%)	0.83	65% (95% CI 0%-90%)	0.58	59% (95% CI 0%-88%)
**eFigure 4A**	0.56	-	0.80	-	0.36	-
**eFigure 4B**	0.56	-	<0.01	-	0.22	-
**eFigure 4C**	<0.01	92% (95% CI 80%-97%)	<0.01	91% (95% CI 77%-97%)	<0.01	90% (95% CI 73%-96%)

CQ-P: Cochran's Q P-value; I^2 statistics for meta-analysis using only two studies are not shown.

© 2022 Lin EPY et al. *JAMA Network Open.*
